# Quality of life in female myocardial infarction survivors: a comparative study with a randomly selected general female population cohort

**DOI:** 10.1186/1477-7525-5-58

**Published:** 2007-10-30

**Authors:** Tone M Norekvål, Astrid K Wahl, Bengt Fridlund, Jan E Nordrehaug, Tore Wentzel-Larsen, Berit R Hanestad

**Affiliations:** 1 Department of Heart Disease, Haukeland University Hospital, Bergen, Norway; 2 Department of Public Health and Primary Health Care, University of Bergen, Bergen, Norway; 3 Section of Health Sciences, Faculty of Medicine, University of Oslo, Oslo, Norway; 4 Center for Shared Decision Making and Nursing Research, Rikshospitalet-Radiumhospitalet Medical Center, Oslo, Norway; 5 School of Health Sciences and Social Work, University of Växjö, Växjö, Sweden; 6 Institute of Medicine, University of Bergen, Bergen, Norway; 7 Centre for Clinical Research, Haukeland University Hospital, Bergen, Norway

## Abstract

**Background:**

A substantial burden associated with MI has been reported. Thus, how survivors experience their quality of life (QOL) is now being given increasing attention. However, few studies have involved women and a comparison with the general population. The aims of this study were to determine the QOL of female MI survivors, to investigate whether their QOL differed from that of the general population, and to evaluate the clinical significance of the findings.

**Methods:**

Two cross-sectional surveys were performed; on female MI survivors and the general Norwegian population. The MI survey included women aged 62–80 years, three months to five years after their MI. One hundred and forty-five women responded, yielding a response rate of 60%. A subset of women in the same age range (n = 156) was drawn from a study of 1893 randomly selected Norwegian citizens. QOL was measured in both groups with the World Health Organization Quality of Life Instrument Abbreviated (WHOQOL-BREF).

**Results:**

The majority (54%) of the female MI survivors presented with ST-elevation in their ECG, 31% received thrombolysis, and 38% had reduced left ventricular ejection fraction. Female MI survivors reported significantly lower satisfaction with general health (p = 0.020) and overall QOL (p = 0.017) than women from the general population. This was also the case for the physical and environmental QOL domains (p < 0.001), but not for the psychological and social relationship domains. Estimated effect sizes between the two groups of participants ranged from 0.1 to -0.6.

**Conclusion:**

The burden of MI significantly affects the physical health of elderly women. Still, female MI survivors fare as well as the general female population on psychosocial QOL domains. Action should be taken not only to support women's physical needs but also to reinforce their strengths in order to maintain optimal QOL.

## Background

Due to better prevention and improved treatment of coronary artery disease (CAD), survival after myocardial infarction (MI) has improved considerably during the past three decades [1]. Although many women survive the acute phase of MI, little is known about their recovery period as mainly studies on recovery after MI are done in men [2,3]. So far, MI has been reported to put a substantial burden on affected individuals by influencing physical as well as psychological, social, economical, and practical aspects of life [3-6]. Still, few studies on MI survivors have focused on the perception of these broad life domains in terms of a multidimensional view of quality of life (QOL). Rather, studies have addressed various unidimensional physical (e.g., symptoms, functional capacity, disease severity); social (e.g., social support); or psychological (e.g., anxiety, depression) aspects of recovery after MI and have labeled these as QOL [7-9]. Consequently, the assessment of global satisfaction with life, as well as broader life domains in female MI survivors, is scant and is thus important to investigate. According to the World Health Organization, QOL can then be defined as *"individuals' perception of their position in life in the context of the culture and value systems in which they live, and in relation to their goals, expectations, standards and concerns" *[10].

QOL measurements aim at assessing treatment efficacy and forming the basis for counseling patients, and establishing new services and health policy decisions. Still, results from QOL studies have not yet been brought to the forefront of these crucial discussions. One reason may be that differences in QOL scores can be difficult to interpret for clinicians and decision makers not familiar with QOL scales and line of research. A consideration of both the statistical and clinical significance of differences in QOL may prove to be valuable. Among the most commonly used distribution-based methods to interpret clinical significance is the effect size [11]. However, studies on QOL in MI survivors have rarely evaluated the clinical significance of their findings [12].

Interpretation of QOL scores may also be enhanced by comparing the scores of a study population with those of a reference population. In comparative studies, it has been reported that female MI survivors have physical, social, and medical disadvantages compared to their male counterparts [7,13,14]. Furthermore, several studies have reported lower QOL in female MI patients than in male MI patients [4,5,15]. The question still remains, would a comparison between sexes supply us with the most valid and relevant information, or would a comparison with the general female population be more feasible? Few such comparative studies have been found [4,12,16-20]. Notably, there were some limitations in these studies as Bengtsson et al. [12] had only 19% women (n = 12), Brink et al [4] 32% women (n = 37), Brown et al. [17] had 34% women (n = 143), and White and Groh [20] had only 27 women participating in their study. Wingate [18] merely compared the QOL data with published norm data, uncontrolled for age or gender, and in the studies of Worcester et al [16] and Claesson et al [19] the disease group was heterogeneous as not all women had experienced MI.

There is clearly a call for a direct comparison between multidimensional QOL of a reasonably sized sample of female MI survivors and that of the general female population of the same age range, as well as a thorough evaluation of the clinical importance of the resulting findings. Hence, the aims of this study were (i) to determine the QOL of female MI survivors, (ii) to investigate whether their QOL differed from that of the general population, and (iii) to evaluate the clinical significance of the findings.

## Methods

### Design

Two cross-sectional surveys were performed; on female MI survivors and the general Norwegian population. Approval from the Regional Committee for Medical Research Ethics, Western Norway, was obtained for the MI survivor survey. The general population survey was also presented to the Regional Committee for Medical Research Ethics, but was considered not to require full formal committee review. Approval from the Norwegian Social Science Data Services was obtained for both surveys.

### Study setting and sample selection

#### Female MI survivors

A sample was drawn retrospectively from patient registers, a database containing ICD-9 codes and demographic data, at one university hospital serving an urban/rural population. The study inclusion criteria comprised the total population of women aged 60–80 years, hospitalized within a 5-year period (1992–1997) and diagnosed with MI (ICD-9 CM code 410). The women were now to be living at home. Those that had other serious illnesses, like cancer or stroke, or those who were cognitively impaired, were disqualified from participating. After controlling for re-admittances and deaths, we totaled up 262 potential respondents. The exclusion criteria was verified through self-report and review of medical records, allowing for 21 women to be excluded because they had other serious illnesses (n = 8), had died (n = 4), were cognitively impaired (n = 4), lived in an institution (n = 2), asserted not to have experienced an MI (n = 1) or their address was unknown (n = 2). The actual number of potential respondents was reduced to 241. A total of 145 women returned the questionnaire, yielding a response rate of 60%. The responders did not differ significantly from non-responders as to age (mean 72.0 vs. 72.8 years, p = 0.154), time since MI (mean 29 vs 31 months, p = 0.496) or length of hospital stay (mean 9 vs. 10 days, p = 0.364).

#### General female population

A representative sample of 4000 randomly selected Norwegian citizens, aged 19–81 years, was invited to participate in the survey. A total of 1893 questionnaires (48%) were satisfactorily completed [21]. Out of these 1893 persons, all women aged 62–80 years were selected, leaving us with 156 respondents from the general population survey for comparison with the 145 female MI survivors. Thirty-four of the 156 responders were late responders. The total response rate for this cohort was 40%. The 231 non-responders in the general female population cohort were significantly younger (mean 70.1 years) than responders (mean 71.4 years) at p = 0.015.

### Instruments

#### Socio-demographic and clinical data

Information on socio-demographic data such as age, educational level, cohabitation and marital status was obtained by self-report in both groups. The MI survivors also reported on chest pain and influence of MI on daily activities. Clinical data on the MI survivors at the time of the index MI were collected by examining all hospital medical records detailing previous angina, previous MI, risk factors (total cholesterol, treated hypertension, diabetes, overweight, family history of CAD and smoking habits), peak creatinin kinase (CK), left ventricular ejection fraction (EF), irregularities in the electrocardiogram (ECG), and treatment given (thrombolysis, percutaneous coronary intervention (PCI), coronary artery bypass grafting (CABG), or medical treatment).

#### The World Health Organization Quality of Life Instrument Abbreviated (WHOQOL-BREF)

The WHOQOL-BREF is an abbreviated 26-item version of the WHOQOL-100. It contains two global items on overall QOL and general health, and four domains: Physical health domain (7 items), Psychological domain (6 items), Social relationships domain (3 items), and Environmental domain (8 items). This generates a profile of domain scores. Each item is based on a Likert scale from 1 to 5. The items ask the respondent "how much," "how often," "how completely," "how good" or "how satisfied" she felt about different aspects of her life in the past 2 weeks. The mean score of the items within each domain is transformed linearly to a domain score scaled in a positive direction from 0–100, such that higher scores denote higher QOL [10].

The instrument has previously been translated into Norwegian according to existing internationally accepted guidelines, and has shown satisfactory results regarding validity and reliability, although the social domain has represented a challenge [21,22]. In the present MI survivor survey, internal consistency measured by Cronbach's alpha ranged from .58 for the social domain to .82–.83 for the other domains. For the general female population cohort, Cronbach's alpha was .58 for the social domain, and ranging from .84 to .87 for the other domains. The instrument has been demonstrated to discriminate between ill and healthy persons [23,24].

### Data collection

The questionnaire was distributed to the MI survivors by a study nurse between December 1997 and January 1998. An introductory letter to potential respondents contained information about the procedure and purpose of the study, and explained that returning the questionnaire would result in inclusion in the study. Hence, returning the questionnaire was regarded as informed consent. Non-responders were reminded once. A pilot study was conducted prior to the main study in order to test the battery of questionnaires by systematically drawing every 11^th ^person on the patient list within a 5-year period. Patients on the list were sorted by year of birth. The pilot study led only to minor changes of the lay-out of the questionnaire; therefore, pilot study respondents were included in the main study.

The survey was mailed to the general population by Statistics Norway from November 2000 to January 2001. Reminders were forwarded once, and returning the questionnaire was considered as informed consent. Early and late responders were recorded, and Statistics Norway supplied information on the age and gender of non-responders [21].

### Data analysis

#### Missing data

We merged the data files of the two surveys, and checked for odd categories and missing data. In the female MI-survivor sample, 56% of the questionnaires had no missing WHOQOL-BREF data, 33% had 1 item missing, 5% had 2 items missing, 3% had 3 items missing and 1% 5 items missing. In the general female population cohort, 93% of the questionnaires had no items missing, 1% had 1 item missing, 3% had 2 items missing. Except for the question on sexual activity which remained unanswered by 38% of the MI-survivors and 3% of the general female population, no single item had more than 6% missing. According to the WHOQOL-BREF manual [10], domain scores cannot be obtained when 20% or more of the items are missing, or when more than two items (or one item in the social domain) are missing in the respective domain. Two female MI survivors and four women from the general population cohort had more than 20% missing and were left out of the analysis. In computation of domain scores, where an item was missing the mean of the other items in that domain was substituted [10]. We also included analyses to characterize those subjects having missing values in one or more item within each WHOQOL-BREF domain with respect to group and socio-demographics (age, education, cohabitation and marital status). No significant difference was found between female MI survivors and the general female population cohort on the rate of missing data in the physical and psychological domains. Multiple logistic regression analysis on the social domain showed significantly more missing data among the female MI survivors (OR = 39.7, p < 0.001), and more missing data among unmarried (OR = 17.3), widowed (OR = 16.7) and divorced (OR = 7.68) compared to married women (p = 0.001). For the environmental domain there was significantly more missing data among the female MI survivors (10%) than in the general female population cohort (3%) (p = 0.013, exact chi square test)). There were no significant relationship between missing data and socio-demographics in the physical, psychological or environmental domains.

#### Statistical analysis

Patient characteristics were compared using the Student's t-test for continuous data, exact Mann-Whitney test for ordinal data, and exact Chi-square test for nominal data. Multiple regression analysis was used to investigate differences between the female MI survivors and the general female population on each domain of the WHOQOL-BREF, adjusting for socio-demographic parameters (age, education, cohabitation, and marital status). To explore whether socio-demographics differentially affected WHOQOL-BREF scores of female MI survivors and those of the general female population, we performed separate regression analysis, including interactions between group and the socio-demographic parameters (age, education, cohabitation, and marital status). Also, to explore possible heterogeneity within the MI group, the regression analyses for the WHOQOL-BREF domain scores were repeated with stratified analyses. Here, the MI-general population dichotomy was replaced by a categorical variable where the MI group was divided into strata, first with respect to EF (normal vs. reduced), and second with respect to time since MI (whole year strata, collapsing all patients with more than 4 years since the MI). For single items, adjusted differences were computed similarly, with confidence intervals computed by bootstrap BC_a _and p-values by permutation tests [25] in order to compensate for the non-normality of the data. Bonferroni correction was applied to all tests on single items within the four WHOQOL-BREF domains in order to correct for the inflating type I error in multiple testing. Therefore, the level of significance was set at p ≤ 0.002 for tests on single items, and p < 0.05 for all other tests. Two-tailed tests were used. The statistical software R (The R Foundation for Statistical computing, Vienna, Austria) was used for bootstrap analyses (R package boot) and permutation tests. All other analyses were performed with SPSS 14.0 (SPSS Inc, IL, USA).

To evaluate the clinical significance of the differences between groups, we computed effect sizes (ES statistic) by dividing the mean difference in scores by the SD of the QOL scores of the general female population cohort [11]. To interpret effect size, we followed the suggestion of Cohen and regarded effect sizes of 0.2–0.5 as small, 0.5–0.8 as moderate, and 0.8 and above as large [11,26,27].

## Results

### Socio-demographic characteristics

The female MI survivors were significantly older than the women in the general population cohort (72 years vs. 70 years, p < 0.001), although the age range (62–80 years) was the same in both groups. Forty-one percent of the MI survivors and 35% of the general female population cohort lived alone. Six percent of the female MI survivors and 12% of the general female population cohort had university education. The two groups did not differ significantly on any of the socio-demographic variables (Table 1).

**Table 1:** Socio-demographics: a comparison between female myocardial infarction (MI) survivors and a general female population cohort

	**MI survivors (n = 145**^†^**)**	**General population (n = 156**^†^**)**	p-value*
**Age**			< 0.001^a^
- Mean	72	70	
- Range	62–80	62–80	
**Cohabitation**			0.342^b^
- Living alone	60	55	
- Cohabitation	85	100	
**Marital status**			0.351^b^
- Divorced	7	9	
- Widowed	62	51	
- Unmarried	6	6	
- Married	68	87	
**Educational status**			0.364^c^
- Elementary school up to 6 years	61	83	
- Elementary school up to 9 years and high school	70	52	
- University/college < 4 years	7	11	
- University/college > 4 years	1	8	

*Significance for differences in proportions by t-test^a^, exact Chi-square test^b^, or exact Mann-Whitney test^c^.^†^n varies between the variables because of missing values.

### Clinical characteristics of the female MI survivors

Female MI survivors differed in time since MI, ranging from 3 months to 5 years. Forty-five percent suffered from angina prior to the MI, and 23% had previously experienced MI. The majority (54%) of the MI survivors presented with ST-elevation in their ECGs. Thirty-one percent of the survivors received thrombolysis. The mean peak CK of the patients was 1099 (range: 28–4270). Thirty-eight percent of the MI survivors had a reduced EF, and two of them had an EF below 30%. In the aftermath of the MI, 38% experienced chest pain and 89% reported that heart disease affected their daily activities (Table 2).

**Table 2:** Clinical characteristics of female myocardial infarction (MI) survivors (n = 145) at index MI

**Clinical characteristics**	**n**^†^**(%)**
**Previous angina**	62 (45)
**Previous acute MI**	32 (23)
**Mean time since MI in months (SD)**	29 (15.9)
**Risk factors**	
- Mean total cholesterol, mmol/L (SD)	7.0 (1.4)
- Hypertension	53 (37)
- Diabetes mellitus	17 (13)
- Overweight	42 (39)
- Family history of CAD	59 (68)
- Smoking habits	
- Non-smoker	68 (55)
- Ex-smoker	21 (17)
- Current smoker	34 (28)
**ECG**	
- Q waves	63 (44)
- ST-elevation	77 (54)
- Left bundle branch block	1 (1)
- ST-depression	47 (33)
- T-inversion	15 (11)
- Normal ECG	6 (4)
**Mean max CK (SD)**	1099 (1000)
**Ejection fraction**	
- 60%	78 (62)
- 30–60 %	45 (36)
- < 30 %	2 (2)
**Treatment**	
- Thrombolysis	43 (31)
- PCI	3 (2)
- CABG	1 (1)
- Medical treatment	92 (66)
Chest pain*	
- Every day	11 (8)
- 3–4 times a week	8 (6)
- 1–2 times a week	16 (11)
- 1–2 times a month	18 (13)
- Seldom or never	87 (62)
CAD affected daily activities*	
- To a very high degree	8 (6)
- To a high degree	24 (17)
- To a certain degree	62 (44)
- To some degree	32 (23)
- Not at all	15 (10)

^†^n varies between the variables because of missing values.

*Self reported at the time of the survey.

CAD: coronary artery disease; CK: creatinin kinase; PCI: percutaneous coronary intervention; CABG: coronary artery bypass grafting.

### Overall quality of life and general health

Female MI survivors were significantly less satisfied with their general health (p = 0.017) and overall QOL (p = 0.020) than women from the general population, as measured by the two global single items in the WHOQOL-BREF (Table 3). Sixty-seven percent of the female MI survivors rated their overall QOL as good or very good compared to 79% in the general female population cohort, while six percent rated their overall QOL as poor or very poor compared to 4.5% in the general female population. As to self-reported health, 16% of the female MI survivors reported being unsatisfied or very unsatisfied with their health compared to 14% of the general female population. The difference between the two groups at the upper end of the scale was larger, as 5% of the female MI survivors were very satisfied with their health compared to 19% of the general female population. In both groups there was a tendency to report better QOL than satisfaction with health.

**Table 3:** Adjusted differences between female myocardial infarction (MI) survivors (n = 141–143) and a general female population cohort (n = 146–152) between scores on global items, quality of life domains, and single items in the WHOQOL-BREF*

	Scores			
WHOQOL-BREF	MI	General population	Adjusted difference (CI)^a^	p-value^b^
**Overall quality of life**	**3.73**	**3.97**	**-0.22 (-0.41, -0.02)**	**0.020**
**Satisfaction with general health**	**3.38**	**3.67**	**-0.27 (-0.49, -0.04)**	**0.017**
**Physical health domain**	**56.7**	**68.9**	**-11.3 (-15.8, -6.9)**	**< 0.001**
Activities of daily living	3.38	3.81	-0.38 (-0.60, -0.16)	< 0.001
Dependence on medical treatment^†^	3.05	2.15	0.92 (0.60, 1.24)	< 0.001
Energy and fatigue	3.19	3.66	-0.41 (-0.62, -0.19)	< 0.001
Mobility	3.34	3.97	-0.56 (-0.81, -0.29)	< 0.001
Pain and discomfort^†^	2.38	2.03	0.39 (0.12, 0.65)	0.002
Sleep and rest	3.25	3.48	-0.18 (-0.45, 0.09)	0.185
Work capacity	3.11	3.55	-0.36 (-0.60, -0.12)	0.001
**Psychological domain**	**66.5**	**69.4**	**-2.0 (-5.6, 1.8)**	**0.262**
Bodily image and appearance	3.52	3.50	0.05 (-0.17, 0.27)	0.629
Negative feelings^†^	2.34	2.18	0.17 (-0.02, 0.37)	0.064
Positive feelings	3.84	3.97	-0.06 (-0.27, 0.13)	0.503
Self-esteem	3.50	3.57	-0.00 (-0.20, 0.19)	0.960
Spirituality/personal beliefs	3.68	3.97	-0.22 (-0.42, -0.02)	0.022
Concentration	3.76	3.84	-0.07 (-0.27, 0.14)	0.508
**Social relationships domain**	**71.1**	**69.3**	**1.4 (-2.5, 5.3)**	**0.457**
Personal relations	4.16	4.08	0.08 (-0.07, 0.25)	0.274
Social support	3.88	3.90	-0.03 (-0.23, 0.18)	0.794
Sexual activity	3.18	3.33	-0.23 (-0.52, 0.05)	0.102
**Environmental domain**	**64.0**	**71.6**	**-6.9 (-10.6, -3.3)**	**< 0.001**
Financial resources	3.71	3.66	0.02 (-0.22, 0.27)	0.859
Physical safety and security	3.63	4.01	-0.35 (-0.54, -0.14)	< 0.001
Access to health care	3.81	3.97	-0.09 (-0.30, 0.14)	0.439
Home environment	4.24	4.28	-0.07 (-0.25, 0.12)	0.444
Information	3.26	3.85	-0.58 (-0.82, -0.34)	< 0.001
Recreation and leisure	2.46	3.21	-0.68 (-0.96, -0.40)	<0.001
Physical environment	3.61	3.92	-0.30 (-0.49, -0.10)	0.001
Transport	3.80	4.03	-0.15 (-0.39, 0.09)	0.191

*Scores on single items range from 1 to 5; a higher score implies better health/quality of life, except for 3 items (†) in which a lower score implies better health/quality of life. Domain scores range from 0 to 100.

^a^Adjusted difference, with 95% CI, based on linear regression for domain scores supplied by bootstrap BC_a _CIs for single items (10000 bootstrap replications stratified by group [MI or general population]). Adjusted for age, cohabitation, marital, and educational status.

^b^Tests based on linear regression for domain scores. For single items, linear regression with permutation tests (10000 random permutations) were used.

### Quality of life domains and single items of WHOQOL-BREF

When examining different areas of QOL, we observed significantly lower scores–implying poorer QOL–in the female MI survivors than in the general female population cohort on the physical health and environmental domains (p < 0.001). Female MI survivors scored significantly lower than the general female population cohort on all physical aspects, except on sleep and rest. The largest adjusted group difference was found with dependence on medical treatment, followed by mobility, and perceived energy and fatigue (p < 0.001). As to the environmental life domain, the groups differed on all items, except for financial resources, home environment, access to health care, and transport. The largest adjusted differences were observed with opportunities for leisure activities and the availability of information needed in daily life (p < 0.001). All single items in these two domains showing a significant difference between the female MI survivors and the general female population cohort were also significant after Bonferroni correction.

The groups did not differ significantly on the psychological domain or on the social relationship domain. As to single items of these domains, there were no significant adjusted differences between the MI survivors and the general female population cohort, except for spirituality and personal beliefs (p = 0.022). This latter item, however, was no longer significant after Bonferroni correction.

We found no interaction when exploring whether socio-demographics had a different effect on the observed WHOQOL-BREF domain scores of female MI survivors compared to those in the general female population. When stratifying the female MI group by EF (60% and above, and below 60%) we found similar results as in the main analyses; the adjusted differences between MI groups and the general female population were significant on the physical health (p < 0.001) and environmental (p = 0.001) domains, and non significant on the psychological (p = 0.402) and social (p = 0.532) domains. Within the MI group, the adjusted difference between normal and reduced EF was non-significant for all domains (all p ≥ 0.291). When stratifying the MI group by time since index MI (in years) we also found results similar to the main analyses; the adjusted difference between MI groups and the general female population were significant on the physical health (p < 0.001) and environmental (p = 0.006) domains, and non significant on the psychological (p = 0.695) and social (p = 0.561) domains. Within the MI groups, none of the stratified groups were significantly different from those with most recent MI (≤ 1 year).

### Clinical significance

**Figure 1:**
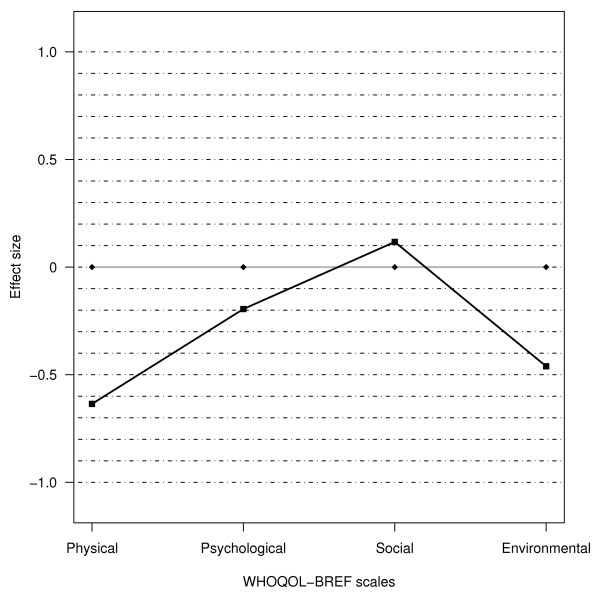
Differences in quality of life scores between female myocardial infarction (MI) survivors (n = 143) and a general female population cohort (n = 152) of the same age group shown as effect sizes. Values less than zero indicate that the quality of life in female MI survivors is poorer than that of the general female population.

The estimated effect sizes of the differences in QOL between the female MI survivors and the general female population cohort were -0.6 for the physical health domain, -0.2 for the psychological domain, 0.1 for the social relationship domain, and -0.5 for the environmental domain (Figure 1).

## Discussion

This study shows that female MI survivors have poorer physical health and score lower on environmental domain items than the general female population. However, MI survivors do not report a worse outcome on psychosocial domains than the general female population. This main finding is rather surprising, because research to date indicates that MI causes not only substantial physical burden but also psychological burden [6].

Our study clearly shows that experiencing an MI limits physical abilities, such as activities of daily living, work capacity, mobility, and energy, and increases pain, discomfort, fatigue, and dependence on medical treatment (Table 3). In this study, female MI survivors experienced significantly more pain and discomfort than did the general female population. In fact, 25% of the female MI survivors experienced chest pain several times a week, and 8% experienced chest pain at least every day. These findings may be related to a low rate of revascularization and use of thrombolysis in these MI survivors (Table 2). Other studies, however, have indicated that angina is a challenge for women post MI [18,28]. In spite of optimal medical treatment and revascularization, some patients still experience pain and discomfort. Therefore, alternative care and treatment should be sought and further work needs to be done to meet the challenges of these patients [29,30]. The lack of energy and fatigue experienced by MI survivors has also been reported by other studies [31]. Motivating patients to participate in rehabilitative activities may help to bolster their energy in every day life. Indeed, cardiac rehabilitation has shown to reduce the burden of MI [32]. Specific programs for women, however, need to be further explored [28,33]. Participation in cardiac rehabilitation programs was unfortunately no option to elderly living in this area at the time of the study. As shown in Table 2, MI clearly had an impact on the daily activities of a majority of these women. Compared to before the MI, 44% of the MI survivors felt that the MI affected their daily activities to a certain degree, while 23% felt that the MI affected their daily activities to a high or very high degree. Notably, 38% of the MI survivors had a reduced EF, suggesting that these survivors had diminished functional capacity. Those having a reduced EF showed a tendency to report lower on the physical health and environmental domains than did the MI survivors with a normal EF, but the adjusted difference was not significant. This difference would be expected to be greater, but may be explained by the fact that only two women had an EF below 30%.

Although the female MI survivors and the general female population cohort showed significant differences on physical health, overall these differences did not affect their state of mind or relationship with other people in a negative way. Women who have undergone MI have comparable levels of self-esteem, satisfaction with their personal relations, and social support received to those of the general female population. This is also true for satisfaction with sexual activity (Table 3). Although research on sexual activity following MI is rather scarce, the existing research base suggests that MI causes uncertainty and reduced libido [34]. Notably, most research has been focused on male erectile dysfunction; female MI survivors have rarely been investigated. The results from the present study suggest that female MI survivors do not have sexual problems that are more extensive than those of women of the same age group from the general population. Although some precaution must be taken because of the high percentage of missing on this item, in the light of previous studies, the result is rather surprising and needs to be further investigated.

One explanation for the high psychosocial scores of female MI survivors may be that the MI made the survivors evaluate and reprioritize their lives, thereby enhancing their psychological well-being. The fact that ill people report similar or even higher psychosocial well-being and QOL than the general population, or even healthy controls, has been shown in a growing number of studies [17,18,35,36]. This has been explained by phenomena like the disability paradox [37] and response shift [38], and possibly also by the sense of coherence [39]. Both the sense of coherence and the disability paradox originates from a health-oriented (salutogenic) perspective, which may well be a path for further investigation. Whether high scores on psychosocial domains may show to be advantageous for the survival of these women is also an avenue for further research.

Female MI survivors in this study reported they experienced sad, anxious, and depressed feelings, but not significantly more often than the general female population. Anxiety and depression after MI has been reported in extensive research [6,40,41]. Studies on women, however, are limited. Still, our psychological domain results also point toward a possibility that positive and negative emotions co-exist. Among the MI survivors, positive feelings received the highest score within the psychological domain. There was no difference between the two groups related to this issue. This observation may prove to be valuable, as the discussion on psychological issues post MI have mainly focused on depression and/or anxiety. Thus, a possible intervention could be also to enhance positive feelings through an empowering dialogue [42].

In the present study, the female MI survivors and the general female population cohort differed significantly on issues included in the environmental domain. It was quite evident that the MI survivors perceived they had less access to the information they needed in their daily lives. Lack of information related to a new life situation after MI has been reported in several studies [43]. For women, this issue may be related to the fact that fewer women participate in rehabilitation programs [28], even though their need for rehabilitation may be greater than in men [44]. Providing patients with the information they perceive to need is an important task of health-care professionals. Information is necessary so that a patient can act in line with post-CAD measures. In the present study, the MI survivors presented with risk factors that should be acted upon (Table 2): 28% were current smokers and 39% were overweight. Moreover, their mean cholesterol level was 7 mmol/L. The fact that 37% were treated for hypertension and 13% were diagnosed with diabetes demand patients to comply with treatment regimens. Secondary prevention, entailing lifestyle changes and complying with medication prescribed, is of utmost importance in order to prevent new cardiac events [45]. Recently, guidelines on cardiovascular disease prevention in women were published [46]. Health-care professionals need to communicate these guidelines to patients.

Interpreting the relevance of QOL data to clinical practice can be challenging. An interpretation of clinical relevance is always subjective, but comparison with a normative population can provide useful information about the impact of MI on QOL as discussed above. To further guide the interpretation of the clinical significance of our findings, we estimated the effect sizes of the difference between MI survivors and the general female population cohort. The effect size of differences on the physical domain was moderate, and that for the environmental domain was moderate to small. We found no statistically significant difference between the groups in the psychological and social domains, and the effect sizes were small or negligible respectively (Figure 1). These negative findings may also show interesting to clinicians and lead us to suggest reinforcement of strengths and positive feelings – possibly through an empowering dialogue – as one path to follow [42]. To advise the patient to increase importance of areas she is doing well and decrease the areas of which are troublesome can be another way to induce positive emotions and enhance QOL [36]. The moderate to small effect size on the physical and environmental domains have clinical relevance because they confirm that MI affects physical aspects of QOL, and indicate that MI survivors perceive to have limited information, less opportunities for recreation and leisure, and feel less physical safe and secure. Motivating patients to participate in rehabilitative activities may mitigate this, in addition to help bolster their energy in daily life and assist them in acting in line with post-CAD measures. Alternative treatment aiming to alleviate chest pain should also be sought. In view of the existing research base presented in the discussion, and in view of our clinical experience, our findings appear to be of clinical importance for the care of female MI survivors. The effect sizes–both the significant and the unexpected negligible–support this assertion.

### Methodological issues

The main strength of the current study is the use of a standardized, validated QOL questionnaire (WHOQOL-BREF) based on a sound definition of QOL. The multidimensional and generic nature of the questionnaire allows for comparison with the general population. Furthermore, the persons in the general population survey were randomly selected, and the persons in the MI survey were the total population of female survivors in a geographical region within a 5-year period. Although the surveys were not performed in the same year, they were performed at the same time of the year.

The response rate of both surveys may possibly limit the generalizability of our findings. A response rate of 60% among the MI survivors is not ideal; thus, the possibility of selection bias cannot be ruled out. However, considering the age of the respondents and the survey being a postal survey, the response rate may be as high as can be expected [47]. Notably, non-responders did not differ from the responders on age, time since MI or length of hospital stay. Since we do not have data on the disease severity of non-responders, there is a possibility of elite bias. We also thoroughly investigated missing values in the data set and generally found the rate of missing values to be low. Few relationships between missing data and socio-demographic characteristics were established. The higher rate of missing data among the currently unmarried female MI survivors on the social domain can be explained by the high rate of missing on the sexual activity item.

The general population survey of Hanestad et al. [21] had a response rate of 48%, while our general female population cohort had a response rate of 40%. This response rate is common in population surveys [48], but low response rates may reduce representativity and produce bias in the data set. The non-responders in the general female population cohort were significantly younger than the responders, but a mere difference of one year can be viewed as less important in this age group. It has been asserted that late responders have the same characteristics as non-responders in population surveys [49]. In an attempt to provide more information on the representativity of the general female population cohort, we explored the possible difference between early and late responders. The late responders did not differ from early responders on any of the socio-demographics or on any of the QOL domains. Therefore, to the best of our knowledge, the non-responders in the general female population cohort did not differ from the responders in any systematic way.

## Conclusion and implications

This study is one of the few studies that directly compare the multidimensional QOL of female MI survivors with that of a general female population cohort of the same age range. It contributes to the knowledge base regarding the recovery period of women after MI through a comprehensive evaluation of the clinical significance of the findings. Our findings indicate that female MI survivors perceive a significantly lower satisfaction with health, score lower on physical and environmental domains, but do not report worse outcome on social relationships or on the psychological domain than women in the general population. Clinicians should recognize that female MI survivors experience fatigue and lack of energy, pain and discomfort, lessened mobility, negative feelings, and have less access to needed information in their daily lives. On the other hand, they also have levels of self-esteem, satisfaction with personal relationships, sexual activity, and social support comparable to other women of their age group. Action should be taken not only to support female MI survivors' physical needs but also to reinforce their strengths in order to maintain optimal QOL. More studies on the recovery period of female MI survivors are needed. In particular, further research is needed in order to identify predictors of QOL and to investigate whether the level of QOL changes during the course of illness. Both qualitative and quantitative approaches are needed in order to elucidate positive and negative emotions after experiencing an MI, and to more fully understand the mechanisms underlying the multidimensionality of QOL.

## Competing interests

The authors declare that they have no competing interests.

## Authors' contributions

TMN designed the study, carried out the female MI survivor survey, collected the medical records data and drafted the manuscript. AKW and BRH carried out the general population survey, and participated in the design of the study. BF participated in the design of the study. JEN participated in the design of the study, and collection of medical records data by reviewing the ECGs. TWL and TMN planned and performed the data analysis. All authors commented on drafts of the manuscript, and read and approved the final manuscript.
